# Do ecological valid stop signals aid detour performance? A comparison of four bird species

**DOI:** 10.1098/rsos.250316

**Published:** 2025-06-18

**Authors:** Anneleen Dewulf, Clara Garcia-Co, Wendt Müller, Joah Robert Madden, An Martel, Luc Lens, Frederick Verbruggen

**Affiliations:** ^1^ Centre for Research on Ecology, Cognition and Behaviour of Birds, Ghent University, Ghent, Belgium; ^2^ Department of Experimental Psychology, Ghent University, Ghent, Belgium; ^3^ Department of Biology, Behavioural Ecology and Eco-Physiology Group, University of Antwerp, Antwerp, Belgium; ^4^ Centre for Research in Animal Behaviour, Psychology, University of Exeter, Exeter, UK; ^5^ Department of Pathobiology, Pharmacology and Zoological Medicine, Wildlife Health, Ghent University, Ghent, Belgium; ^6^ Department of Biology, Terrestrial Ecology Unit, Ghent University, Ghent, Belgium

**Keywords:** response inhibition, stop-signal detection, comparative approach, birds, detour

## Abstract

Response inhibition (RI), or the stopping of actions, is considered a key component of flexible and adaptive behaviour. Across fields, RI is often treated as a unitary cognitive mechanism. However, we propose that RI consists of a chain of cognitive processes, including the detection of a stimulus, the selection of an appropriate behaviour (go or stop) and the implementation of it (execution or inhibition of a motor response). From this, we propose that individual variation in RI can arise at the early signal detection stage. This idea was tested in a detour barrier task, which is one of the most popular tools to study RI in non-human animals. The role of signal detection in detour tasks has been largely neglected, with a few notable exceptions. We therefore partially replicated two previous studies that manipulated the perceptual characteristics of the barrier, while addressing some conceptual and methodological shortcomings of the original work. Specifically, we compared how detour performance of four bird species (i.e. white leghorn chickens, Japanese quails, herring gulls and domestic canaries) is differently influenced by vertical-bar (VB) and horizontal-bar (HB) barriers. In contrast to the previous work, performance on the detour task did not improve when the perceptual characteristics of the barrier matched the ecological niche of the species. However, all species showed some level of learning, as evidenced by shorter detour latencies (except in herring gulls) and fewer persisting attempts. These findings highlight the need for replication studies and emphasize the importance of improving methodological and conceptual design factors to further investigate the underlying mechanisms of RI in animals. Preregistered Stage 1 protocol: https://osf.io/qvxgh (date of in-principle acceptance: 20/03/2023).

## 1. Introduction

Response inhibition (RI) refers to stopping or cancelling actions that are no longer relevant, inappropriate or overly risky [1,2]. It is often regarded as a critical component of flexible and adaptive behaviour [2]. For example, animals living in urban environments must often inhibit no-longer relevant behaviours when confronted with environmental conditions that differ significantly from their ancestral ones [3]; lower-ranked animals need to inhibit inappropriate, disobedient behaviour in the presence of dominant animals [4]; and foraging animals must refrain from approaching a food source when this action becomes overly risky due to the emergence of a predator [5]. These examples demonstrate that RI (or a lack thereof) can have important fitness consequences (e.g. the animals may be predated if they fail to stop foraging when the predator emerges).

One of the most popular tasks to study RI in animals is the detour task [6–13]. In this task, the direct path to a motivationally salient stimulus (e.g. food or a social companion) is blocked by a barrier or cylinder. Animals have to inhibit their prepotent response to go directly for the reward (as the direct path is blocked) and instead make a detour around the barrier or cylinder to obtain the reward. Detour tasks have been used in non-human animals, such as birds, to study, for example, how the social or physical environment shapes RI. For example, wild Australian magpies (*Gymnorhina tibicen*) demonstrated superior detour performance when reared in large compared with small social groups [14]. Another study found that pheasants (*Phasianus colchicus*) showed superior detour performance when reared in spatially unpredictable compared with predictable environments [15]. Combined, these findings suggest that RI development is facilitated in, for example, environments with high social demands or environments that promote the expression of diverse foraging strategies.

Typically, performance in the detour task has been linked to the variation in the effectiveness of a single cognitive control function, ‘response inhibition’, or more generally, ‘inhibitory control’ (which is an umbrella term for various types of inhibition, which may or may not be related to each other [16]). However, by referring to general ill-defined cognitive constructs such as RI (or even worse, a general umbrella term such as ‘inhibitory control’), we do not explain the underlying cognitive mechanisms or building blocks of RI [17], as the explanation is ‘just as mysterious as the thing it is supposed to explain’ [18]. To address this issue in the human RI literature, a theoretical framework of RI has been proposed [17]. Based on empirical work in humans, primates, and rodents, the authors of the framework proposed that RI involves a chain of processes. More specifically, RI would involve at least three basic processes: the detection of a ‘stop signal’ (detection), the stochastic accumulation of information (selection) and suppression of the motoric output (implementation). Furthermore, these core processes can be modulated by a set of processes that take place on shorter (seconds, minutes, hours or days) and longer (months or years) timescales. Depending on the species, this can involve for example outcome monitoring, anticipatory adjustments and both short-term and long-term learning. Here, we argue that some of these cognitive processes play a role in RI across species (without assuming a one-to-one mapping for the full processing chain). In particular, in the present study, we propose that one of these core processes, namely stop-signal detection, is a crucial (but largely ignored) building block of RI across species, including avian species.

## 2. The crucial role of stop-signal detection

Several lines of evidence indicate that signal detection may play a critical role in RI, particularly in humans and non-human primates. For example, several behavioural studies reveal that RI is impaired when visual distractors occur in the environment [19], or when stop signals are hard to perceive [20]. Neurophysiological and computational work also demonstrated that early perceptual processing of potential stop signals (which could be for example an obstacle, or in case of humans, a red light) determines to a large extent whether individuals can successfully inhibit a response or not [21–25].

Thus, it appears that RI may largely depend on the outcome of perceptual processes. However, the crucial contribution of these processes to successful RI is rarely acknowledged or studied in the non-human animal cognition domain, with a few notable exceptions. For example, researchers found that avian RI was improved when the visibility of a stop signal (i.e. a predator) was improved (e.g. when the predator occurred against a white background, in bright light, or in short grass) [26,27]. Other studies suggested that RI in the detour barrier task is affected by the perceptual characteristics of the barrier (i.e. the type of stop signal). For example, Regolin *et al*. [9] (Exp 1) exposed 2 day old white leghorn chickens (*Gallus gallus domesticus*) to a variety of barrier types. These included a barrier with vertical bars or stripes (VB), and a barrier with horizontal bars (HB). The authors found that RI performance was impaired (i.e. the time required to successfully detour around the barrier) when faced with VB barriers compared with HB barriers. Both VB and HB barriers occluded the reward behind the barrier to a similar degree (i.e. 20% compared with a fully transparent barrier). Thus, the differences between these two barrier types cannot be attributed to differences in physical reward occlusion. Instead, the authors suggested two alternative potential explanations for this asymmetrical effect, namely (i) the degree of subjective occlusion and (ii) the ecological validity of stop signals.

First, despite equal reward occlusion by each barrier type, the behavioural repertoire of ground-moving animals consists primarily of horizontal movements (e.g. walking, running). Consequentially, these animals can ‘subjectively’ perceive a reward as less occlusive (i.e. more visible) with VB than HB barriers (making it harder to inhibit the response to go directly for the reward) [28]. However, follow-up experiments in which the occlusion of the reward was directly manipulated were inconsistent with this ‘subjective occlusion’ account [9]. Second, differential performance between VB and HB barriers might be due to the ecological niche of the species. Gallinaceous birds such as chickens are mainly terrestrial animals that have occupied niches that consist of penetrable long grass and twigs. Regolin *et al.* [9] therefore argued that it might be harder for gallinaceous birds to detour around VB than HB barriers, as the former would mimic the penetrable vertical vegetation of their ecological niche (whereas in the detour task, the VB barrier is of course, not penetrable).

Zucca *et al.* [12] further investigated this ecological-niche hypothesis by comparing detour performance in another gallinaceous bird species, hybrid (Japanese) quails (*Coturnix coturnix × C. japonica*), with performance in two species with a substantially different ecological niche, namely yellow-legged gulls (*Larus michahellis*) ^1^ and domestic canaries (*Serinus canaria*). They used a variant of the detour task with multiple compartments and again, VB and HB barriers (14% reward occlusion compared with a transparent barrier^2^). In this study, the authors considered both probability of a correct response (i.e. going to the correct compartment during their first attempt) and the latency to detour as measures of RI. They found that the detour accuracy for quails was lower (i.e. RI performance was impaired) for VB than for HB barriers. This seems consistent with the findings of Regolin *et al.* [9], although it should be noted that Zucca *et al.* [12] only found a significant effect for detour accuracy, but not for the latency to detour, the measure of RI in the study of Regolin *et al.* [9]. For yellow-legged gulls, detour accuracy was not influenced by barrier type, but detour latency was. Specifically, the latency to detour was longer (i.e. RI was impaired) for HB than VB barriers. Again, the authors attributed this to the species’ ecological niche. Specifically, Zucca *et al*. [12] argued that in the (original) ecological niche of young, yellow-legged gulls, chicks are accustomed to consider the vertical ground vegetation of sand dunes as largely impenetrable. According to the authors, it might therefore be harder for (young) gulls to detour around horizontal than vertical barriers, as the latter would be perceived as less penetrable (note that Zucca *et al.* [12] tested juvenile gulls that could not fly yet). Lastly, canaries were unable to detour around the barrier, although they made several attempts to fly over the barrier demonstrating that they were sufficiently motivated. Zucca *et al.* [12] therefore hypothesized that the detour task is not considered to be a real problem for the two-month old canaries. The authors suggested that, after all, canaries are aerial birds, allowing them to tackle obstacle problems by simply flying over them (but which was not possible in the detour task due to the dimensions of the used apparatuses).

In sum, the results of Regolin *et al.* [9] and Zucca *et al.* [12] indicate that the characteristics of the ‘stop signal’ matter in the detour task, potentially shedding new light on RI in avian species. However, some concerns can be raised about certain features of the previous studies (which are summarized in table 1). First, the sample size was low (at least for some species), the studies (inconsistently) used within- and between-species designs, the number of trials per barrier type differed within and between species and the number of sessions per barrier type fluctuated between species (e.g. yellow-legged gulls received three sessions per barrier type spread over 3 days, while hybrid quails received one session per barrier type). The latter two issues are also problematic from a conceptual point of view, as previous work indicates that learning will influence RI [29,30], including in the detour task [10]. Second, both studies used less-common variants of the detour task, which complicates comparisons with the wider literature. Additionally, they used less common, hard to standardize (social) rewards, which complicates between-species comparisons of RI behaviour. Similarly, the large differences in developmental trajectories and the lack of adapting the test apparatus to the morphological differences between species, also complicated the between-species comparisons.

**Table 1.** Methodological features of Regolin *et al.* [9], Zucca *et al.* [12] and the present study.

source	Regolin [9]	Zucca [12]			current study
1. methodology					
species	white leghorn chicken	hybrid quail	yellow-legged gull	canary	all four species
design	between	between	within	within	mixed
total sample	750 (250)^a^	90	5	26	240 (60/species)
sample per barrier type	102 (34)^a^	18	5	26	60/species
trials per barrier type	1	10	10	1 or 10^b^	3
sessions per barrier type	1	1	3	1	1
2. detour task	two compart.	four compart.	four compart.	four compart.	simple
3. reward	cagemates	reflection	reflection	reflection	food
4. baseline covariate	no	no	no	no	yes
5. DV's	latency	latency accuracy	latency accuracy	latency accuracy	latency persistence
6. enclosure					
social density	3	1	5	5	± 10
fence	NA	vertical	bricks	vertical	mesh netting
7. test age	2 days	1 M	10−25 days	4−6 M	species specific
8. apparatus					
test box: L × W	120 × 35	150 × 75	150 × 75	150 × 75	scaled/species
test box: H	60	40	40	40	barrier H
barrier-entry distance	27	27	27	27	scaled/species
barrier: L × H	10 × 20	23 × 26	23 × 26	23 × 26	scaled/species
barrier line: W	0.3	0.2	0.2	0.2	scaled/species
gap between barrier lines	1.2	1.25	1.25	1.25	scaled/species

DV’s, Dependent variables.

^a^ Animals were reared and tested in groups of three. The means of each trio was used as individual data for the subsequent analysis.

^b^ 6/26 canaries received 1 trial/barrier, 20/26 canaries received 10 trials/barrier. Measurements are in cm.

## 3. A partial replication

To prove the significance of the previous study, our study investigated the role of stop-signal detection in avian RI by partially replicating the studies of Regolin *et al*. [9] and Zucca *et al*. [12]. The importance of the original studies is indubitable, as they are one of the few studies that aimed to ‘deconstruct’ avian RI performance by focusing on the underlying cognitive processes (in this case, stop-signal detection). Additionally, Zucca *et al*. [12] implemented a comparative approach to investigate whether differences in how the stop signal might be perceived by different species could contribute to variation in RI.

**Figure 1.**
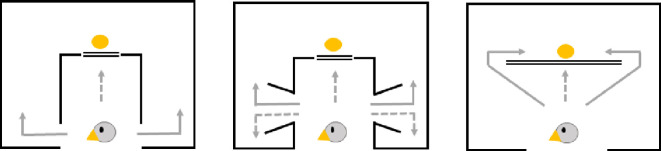
A display of the three detour task variants: the two-compartment detour task (left [9]), the four-compartment detour task (middle [12]) and the simple detour barrier task (right, current study). Double line: barrier; full arrows: correct responses; dashed arrows: incorrect responses.

In our partial replication, we made several changes to address commonly raised concerns in the detour literature (including the concerns raised in the previous section, see table 1). First, we directly compared four species (white leghorn chickens, Japanese quails, herring gulls [*Larus argentatus*^3^] and domestic canaries), in a well-powered mixed design analysis with *Species* as between-species factor and *Barrier* (VB vs. HB barrier) as within-species factor. Hereafter, each species will be referred to by its common name for clarity: chicken (excluding ‘White Leghorn’), quail (excluding ‘Japanese’), gull (excluding ‘herring’) and canary (excluding ‘domestic’). All species were given an equal amount of trials and sessions per barrier type (see below). Second, the perceptual characteristics of the barrier (i.e. VB vs. HB barriers) were manipulated in a simple detour barrier task (which is the most common variant of the detour problem [28], rather than a four- [12] or two-compartment [9] detour task). See figure 1 for an overview of the designs. Third, the unconditional reward was food instead of a social stimulus (as in Regolin *et al.* [9] and Zucca *et al*. [12]). Food is a common reward in laboratory tests and has a high incentive value across species and individuals. Furthermore, its subjective value can be better standardized both within and between species compared with social rewards. Fourth, non-cognitive, motivational states can influence detour performance [11,31]. Therefore, we collected for each individual a ‘multi-baseline’ measure of their general motivational state (which could be a combination of, for example, non-transparent obstacle neophobia, test box neophobia, food motivation or motivation to explore). This ‘multi-baseline’ measure was obtained with an opaque barrier during habituation (see below). We included this as a covariate in our statistical models to increase the likelihood of detecting barrier type effects within species conditional on/adjusted for the ‘multi-baseline’ measure of an individual’s general motivational state.^4^ Fifth, our study considered two measures of interest, namely the latency to detour [9,12] and the time spent in proximity to the barrier (persistence). The last variable was not included in the original studies but adds substantial information about variation in (un)successful inhibition following the different barrier types. Note that this measure also captures ‘accuracy’, as all birds that did not peck at the barrier (i.e. an accurate response) got a score of 0, whereas all birds that pecked got a score > 0. Sixth, for all species, (fledged) chicks were raised in groups of approximately 10 individuals, as variations in detour performance had already been reported with fluctuating social group sizes [14]. Furthermore, mesh netting was used for the enclosures of all species (preventing variation in experiences with (im)prenetrable VB or HB objects in the enclosures). Seventh, detour performance of the different species was compared when they are on similar levels in their developmental trajectory (see, for example, Kabadayi *et al.* [6, 31], and Verbruggen *et al.* [17] for the influence of cognitive maturation on RI), and again, with similar experiences in the enclosure, keeping in mind the precocial-altricial spectrum (see below). Finally, the size of the test apparatuses and barriers (including the width and the in-between line gaps) was scaled based on the morphological characteristics of each species (see below). For example, Zucca *et al.* and [12] used the same test box for all three species and argued that this was appropriate because they tested species at different ages. Nevertheless, one could still expect substantial differences in body size (e.g. a one-month old quail is substantially larger than a two-month old canary). As (relative) distance towards the reward influences detour performance [28], it was therefore important to control for this as well.

## 4. Predictions

First, we predicted better RI performance for ecologically valid compared with non-valid stop signals, as should be reflected in shorter latencies to detour and less time spent persisting. As the (original) ecological niche of our species substantially differs (chicken and quails: penetrable vertical terrestrial vegetation; gulls: impenetrable vertical vegetation of sand dunes; canaries: aerial environment), the ecological validity of stop signals would be species-specific. Specifically, for chickens and quails, we expected better detour performance for HB compared with VB barriers (thus, detour performance HB > VB). We expected the opposite pattern for gulls (i.e. detour performance HB < VB). Finally, based on the findings of Zucca *et al.* [12], we did not expect differences between VB and HB barriers for canaries (i.e. detour performance HB = VB). Overall, this pattern should result in a statistical interaction between *Barrier* and *Species* (*Prediction 1*).

Second, as each session consisted of three trials (of the same barrier type), we also looked at how detour performance improved within each session. Based on previous studies, we predicted that detour performance should improve across trials within a session (*Prediction 2*).

Furthermore, we explored if the learning effect (i.e. improved detour performance across trials) interacts with the ecological validity of the stop signals. There are two possible patterns that would result in a three-way interaction between *Species*, *Barrier* (HB vs. VB barriers), and *Trial* (1–3) (*Explorative Prediction 3*). First, detour performance might be better for ecologically valid compared with non-valid stop signals at the beginning, but this pattern might diminish over time as individuals learned to stop (i.e. the differences between barrier types would decrease). Second, detour performance might be poor at the beginning for both barrier types, but learning to stop might be easier for ecologically valid signals compared with non-valid stop signals (i.e. the differences between barrier types would increase). Both patterns would be theoretically meaningful, but we did not have *a priori* predictions about the direction of the three-way interaction.

## 5. Methods

We report how we determined our sample size, all data exclusions, all inclusion/exclusion criteria, whether inclusion/exclusion criteria were established prior to data analysis, all manipulations and all measures in the study.

### 5.1. Subjects and housing

Quails, gulls and chickens were raised and tested at the avian research facilities of Ghent University (Lab number LA1400452), located at the Wildlife Rescue Center (WRC) in Ostend (Belgium). The canaries were raised and tested at the avian research facilities of the University of Antwerp (Lab number LA1100161) in Wilrijk (Belgium).

#### 5.1.1. Sample size

We originally registered to test 60 individuals per species. *A priori* power sensitivity analyses done in G*Power [32] indicated that this was sufficient to detect small effects; it was also the largest number that was practically feasible.^5^ For the sensitivity analysis, we used a mixed ANOVA model with one between-subjects factor (four levels; corresponding to our *Species* factor) and two within-subjects factor (one with two levels—*Barrier*—and one with three levels—*Trial*). This indicated that our sample size of 60 animals per species (240 in total) was sufficient to detect a *Species* × *Barrier* interaction effect (*Prediction 1*) with a small effect size (Cohen’s *f* effect size of 0.12; [34]; Power = 0.80; cor. among RM = 0.5; we used an alpha of 0.025 to correct for the fact that we had two dependent variables measuring (slightly) different aspects of detour performance). Second, our sample size was sufficient to detect a small effect of *Trial* (*Prediction 2*; Cohen’s *f* effect size of 0.09 [34]). Third, our sample size was sufficient to *explore* a small effect (Cohen’s *f* effect size of 0.09 [34]) for the *Species* × *Barrier* × *Trial* interaction effect (*Explorative Prediction 3*). Due to higher than expected post-hatch mortality, the quail sample size was slightly reduced to 58. This reduction did not result in meaningful differences in effect size for our three predictions.^6^

Our sensitivity analyses were based on mixed ANOVAs (fixed-effects models with between- and within-species factors). However, as discussed below, we analysed our data with (G)LMMs, which are currently not covered by G*Power or most other power-estimation tools. These mixed-effect models are more flexible in assigning variance as they allow for the specification of both fixed and random effects. However, by accounting for unexplained variance (see below), our proposed mixed effect models are more powerful than the fixed-effect model ANOVAs used in our sensitivity analyses (and than the models used in the studies of Regolin *et al.* [9] and Zucca *et al.* [12]). Thus, the sensitivity analyses discussed here are a conservative estimate.

#### 5.1.2. White leghorn chickens and Japanese quails

Chicken and quail eggs were obtained from local breeders in Belgium. At the WRC, the eggs were incubated in Brinsea Ova-Easy incubators (temperature = 37.5${,}^{\circ }$C; humidity = 45% for first 15 [quail] or 17 [chicken] days, after which humidity = 70% till hatching). Once hatched, chicks received a unique colour ring combination prior to being housed in groups of ± 10 chicks^7^ per indoor enclosure [size = 1 m × 1 m × 2 m; L × W × H]; ambient temperature = 15–25${,}^{\circ }$C; humidity = 40–80%; photoperiod = 14 : 10 L : D; type of wire fencing = mesh netting). Birds were *ad libitum* provided with a chicken meal mixture (Aveve Chicken Start Mash) and water. Shelter, additional heating panels (30 × 30 cm; till Day 7), and pecking objects (pine cones) were available. The (precocial) chickens and quails were tested at ± 3 weeks (see below for justification of species-specific test age). After testing, the individuals were euthanized by certified staff.

#### 5.1.3. Herring gulls

Gull eggs were collected by the ‘Agentschap voor Natuur en Bos’ and the gull patrol team in Ostend (https://www.oostende.be/meeuwen) who are authorized to remove gull eggs along the Belgium coast for various reasons. The eggs were collected prior to pipping and were safely transported to the WRC. At the WRC, the eggs were further incubated in Brinsea Ova-Easy incubators (temperature = 37.5${,}^{\circ }$C; humidity = 45%) and checked twice per day for signs of pipping. When gull embryos reached the pipping stage, they were placed in a hatchery (temperature = 37.2${,}^{\circ }$C; humidity = 50%). Once hatched, the semi-precocial gull chicks received a unique colour ring combination prior to being placed in boxes with netting bottoms (size = 1.20m × 0.60m × 0.60 m; L × W × H) within heated rooms (ambient temperature = 15–25${,}^{\circ }$C; humidity = 40–80%; typical photoperiod = the natural photoperiod at the latitude of Belgium; type of netting = grid) for 5 days (and till their body mass exceeded 60 grams). During this period, the gulls were hand-fed small pieces of fish and dog pellets (soaked in water), supplemented with Akwavit (Kiezebrink Focus on Food, The Netherlands). We also provided one heating panel per box. After this initial indoor period in the boxes, the gull chicks were housed in groups of ±10 individuals^7^ per outdoor enclosure (size = 5 m × 1.95 m × 2.65 m; L × W × H), type of wire fencing = mesh netting, with an extra heating panel for the first couple of days (note that the exact number of days depended on the weather conditions). Food (a mixture of 75% dog food soaked in water and 25% defrosted fish, supplemented with Akwavit) was provided four times per day (the default policy at the WRC); water was provided *ad libitum*. The (semi-precocial) gulls were tested when they were approximately three weeks old (see below). After testing, gulls were moved to large flight cages to dehabituate them from human handling (and hence improve their survival rates). They were released into the wild when they were approximately 8−10 weeks old.

#### 5.1.4. Domestic canaries

*Domestic* canaries (of the Fife Fancy type) were obtained from long-term, breeding populations at the Department of Biology (‘Behavioural Ecology and Ecophysiology’ research group) of Antwerp University. Canaries are altricial species, and nestlings are thus highly dependent on their parents for food. Therefore, chicks were only separated from their parents at the end of the nestling period (i.e. when they were ± 25 days old) (GarciaCo *et al.*, 2024, unpublished data).^8^ At this point, the canaries were moved in groups of ±10 individuals^7^ to indoor aviaries of Antwerp University (size : 1 m × 2 m × 2 m; L × W × H; ambient temperature = 15–25${,}^{\circ }$C; humidity = 40–80%, photoperiod = the natural photoperiod at the latitude of Belgium; type of wire fencing = mesh netting). The canaries were marked with a permanent marker for individual recognition at hatching and ringed with a closed metal ring when their body mass exceeded the predetermined threshold of 7 g. Upon arrival at the indoor aviaries, canaries received a unique number-colour ring combination (the default policy at the University of Antwerp). In the aviaries, canaries were provided with canary seed mixture and egg food (van Camp, Belgium), water, shell grit and cuttlefish bone *ad libitum*. They were tested at seven weeks (approximately three weeks after fledging; see below). After testing, canaries returned to their local breeding population in the University of Antwerp.

#### 5.1.5. A comparative testing age

Our previous work [36] indicates that three weeks is an ideal testing age for large gulls (incl. herring gulls) in detour tasks and other related cognitive tests. Gulls are semi-precocial, but only require handfeeding for the first couple of days (and most start eating independently after two/three days). Furthermore, the gull chicks can already move around (and explore their environment) from Day 1. Chickens and quails are precocial, which implies that they can feed independently and explore their environment from Day 1. Given the overall similarities, we therefore tested chickens, quails and gulls when they were approximately 3 weeks old (i.e., habituation happened on days 16−18; testing happened on ≈ days 19−20). By contrast, canaries only become independent when they are approximately 25 days old (see previous subsection). At this point, they were moved to larger enclosures and housed in groups. To ensure that the altricial canaries had a similar (15 day) experience with their enclosure and their cagemates as the (semi-)precocial species, habituation and testing of canaries was delayed with 25 days (i.e. habituation happened ≈ on days 41−43; testing on ≈ days 44−45)

### 5.2. Apparatus

For each species, the test apparatus consisted of a two door start box, a test box, a barrier and a feeding bowl. Performance of the birds was monitored using a camera placed centrally at the top of the testing arena (Sony Action Cam HDR-AS50). In the test box, a VB or HB barrier blocked the direct path to the unconditional reward (i.e. the food in the bowl) that was immediately placed behind the barrier. The species-specific unconditional food reward (chickens and quails: chicken meal, gull: dog pellets and fish, canaries: canary seed mixture and egg food) consisted of clearly visible food, placed in a coloured bowl. For chickens and quails, these were coloured green and yellow (brand: Junai, The Netherlands); for gulls and canaries, these were coloured orange-brown (brand: Elho, Belgium).^9^ To avoid satiation after the first trial on test trials (see next section), the pile of food was largely covered with a transparent perspex cover, with only a small bit of accessible food placed on top of the cover. The VB and HB barriers were made of transparent perspex on which 18 vertical and horizontal lines, respectively, were painted per species (see below). To prevent canaries from flying over the barrier (as an alternative way of avoiding the barrier), floor-to-ceiling barriers were used for all species.

**Figure 2.**
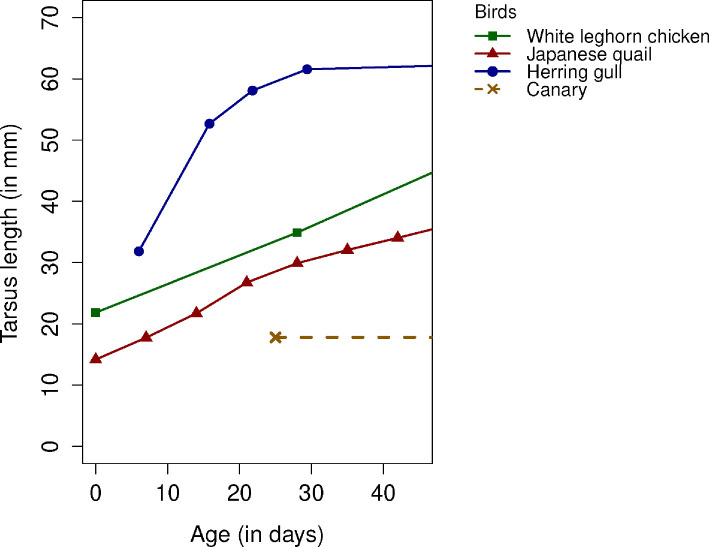
Full lines: reported tarsus growth during early life for white leghorn chickens [37], Japanese quails [38] and herring gulls [36]. Dashed lines: hypothetical tarsus length for canaries based on the assumption that tarsus length does not change (much) after fledging (Garcia-Co *et al*., 2024, unpublished data).

The size of the test apparatus was adjusted per species. In a recent study from our laboratory, we tested gulls in a detour task (akin to the task proposed here, but with transparent and non-transparent barriers). In this study, the starting box was 35 × 20 × 26 cm (L × W × H) and the test box was 145 × 88 × 132 cm (L × W × H). The barrier was 40 × 40 cm (L × H) and was placed 50 cm from the start box entrance (with approximately 24 cm between the edges of the barrier and the sides of the test box). In the present study, we used the same set-up for the gulls, and re-scaled all values based on tarsus length at testing age (see table 2 for the values for each species). For the chickens, quails and gulls we used the growth curves (figure 2) for tarsus length reported in previous studies [36–38]. For canaries, no such growth curves were available. However, a recent study (Garcia-Co *et al*. 2024, unpublished data) measured tarsus length at day 25. Given that morphological traits (incl. tarsus) seem to plateau at a similar moment in the lifespan of a canary, we used this tarsus measure at day 25 as our measure for the tarsus length at testing age. In addition, the black painted barrier lines (18 in total per species) occluded the food reward by approximately 14% [12]. As a consequence, the width of the barrier lines (and of the in-between gaps) as described in the study by Zucca *et al.* [12] was adjusted to the re-scaled barrier size dimensions per species (see table 2).

**Table 2.** The upper table shows the tarsus length and species-specific re-scaled test apparatuses based on the herring gull detour set-up of Troisi *et al.* [36]. The lower table shows the re-scaled width of the barrier lines (and of the in-between gaps) for each species based on the detour set-up of Zucca *et al.* [12].

	white leghorn chicken	Japanese quail	herring gull	fife fancy canary
source	Yeasmin [37]	Dudusola [38]	Troisi [36]	(Garcia-Co 2024 unpublished data)
test age (days)	19	19	19	44
sample size	130	2591	42	69
mean tarsus (mm)	30.71	25.32	55.56	17.81
ratio	1.81	2.19	1	3.12
apparatus (rescaled)				
start box: L × W	19.35 × 11.05	15.95 × 9.11	35 × 20	11.22 × 6.41
test box: L × W	80.15 × 48.64	66.08 × 40.10	145 × 88	46.48 × 28.21
barrier-entry distance	27.64	22.79	50	16.03
barrier: L × H	22.11 × 22.11	18.23 × 18.23	40 × 40	12.82 × 12.82

	white leghorn chicken	Japanese quail	herring gull	fife fancy canary
barrier line: W	0.17	0.14	0.31	0.10
gap between barrier lines	1.06	0.87	1.91	0.61

The estimated tarsus length at testing age for (semi-)precocial species is derived from a linear equation using the two nearest measuring points for a mixed-sex sample (except for white leghorn chickens, where an additional average was calculated over pullets and cockerels). For re-scaling the test apparatuses, the unrounded factor per species was utilized. Unless specified otherwise, measurements are in cm.

### 5.3. Procedure

Prior to the start of the experiment, birds were habituated for 10 days in their enclosure to feed from a coloured food bowl, which was identical to the bowl used during both habituation and testing in the test box. For all species, the feeder was placed on the ground, to simulate ground feeding during the test. When they reached the appropriate age (see above), animals were tested for 5 consecutive days (i.e. 3 habituation and 2 testing days). Food in the enclosures was provided *ad libitum*, but the evening before an individual’s habituation or testing day, the feeders were removed from the enclosures at 18.00 (after the last feeding time). This created a non-feeding period during the night (which is normal and also happens in non-experimental conditions), followed by (shortly) delayed feeding in the morning to prevent birds from overindulging prior to habituation or testing. This is in line with other studies using the same species (chicken: e.g. [39]; quail: e.g. [40], and unpublished data from our laboratory on gulls: e.g. [Dewulf, Knoch, *et al.*, 2025, unpublished data]; canaries: e.g. [41]). After all individuals of one enclosure completed the habituation or testing trials for the day, food was again provided *ad libitum*.

On the 3 habituation days (08:00–10:30), each bird received 1 trial per day where it could freely explore the test box and feed from a centrally placed coloured food bowl. During the second and third habituation day, an opaque barrier was placed just behind the coloured food bowl. This allowed us to obtain a ‘multi-baseline’ measure of an individual’s general motivational state (which could be a combination of, for example, non-transparent obstacle neophobia, test box neophobia, food motivation, motivation to explore; see below). The current habituation set-up (i.e. the food bowl *in front* of the barrier) was designed in such a way that acquiring a motor routine during habituation was unnecessary and could not confound subsequent detour performance with the barred barriers [10].

Each bird participated in one session per day on the 2 testing days (10.30–14.30). Each session consisted of three trials with one barrier type. The order of barrier type (i.e. HB or VB barrier) was pseudo-randomized within and between species, across the 2 testing days.

Due to the natural breeding season of the wild gull and the canary, birds hatched non-simultaneously. In order to guarantee an appropriate test age (see above), we grouped individuals of a similar age per enclosure; and then habituated or tested birds per enclosure (by taking into account the average age of the enclosure). Although there was no fixed breeding season for quails and chickens, incubation happened in ‘batches’ (due to reduced egg production/supply). As a result, an identical grouping procedure within these species was applied.

At the beginning of each trial, each bird was gently placed in the dark two-door start box. The trial started when the researcher opened the first non-transparent cardboard door of the start box. This permitted the bird to see the test arena but not access it. After 15 seconds, the second, transparent door of the start box was opened and the bird could enter the test box. If the bird did not exit the start box within 30 seconds, it was gently pushed forward (by sliding the back of the starting box forward [36]). The habituation trials ended when the individual ate from the food bowl for 30 seconds or when the maximum trial time had been reached (i.e. 5 minutes and 15 seconds). The test trials ended immediately when the individual ate from the food bowl (to avoid food satiation on subsequent trials) or when the maximum trial time had been reached (i.e. 2 minutes and 15 seconds). Maximum trial times during habituation were longer than during testing, as the main goal of the habituation was to familiarize each bird with the test material (and obtain a ‘multi-baseline’ measure of an individual’s general motivational state). The maximum duration of a test trial was 2 minutes (after an additional 15 seconds inside the start box with the second, transparent door), which is in line with other studies (e.g. [31] and [42]). Two minutes should be sufficient, especially because our barriers were not entirely transparent (hence, partially occluded the food reward), making it easier to execute a detour response [28].

Gulls were tested during the second half of June 2023 and 2024 (restricted to breeding season), quails in November 2023 (autumn), chickens in February 2024 (late winter) and canaries in May 2024 (late spring, again, restricted to breeding season).

## 6. Data processing and analysis

### 6.1. Video recording and analysis

**Figure 3.**
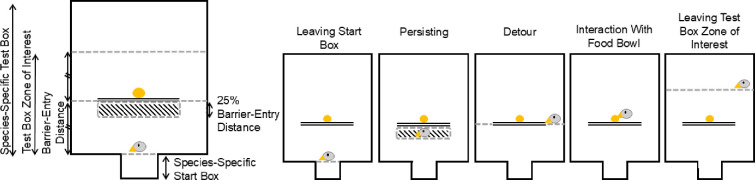
Visualization of the species-specific dimensions (left) and the behaviours (right) that were coded in BORIS. Double line: Barrier; hatched area: species-specific 'barrier zone of interest'; dashed lines: (fictitious) lines that needed to be crossed by the bird, see table 3.

The videos of the second and third habituation trials and the three test trials per test session were coded using the free, open-source ‘Behavioural Observation Research Interactive’ Software (BORIS, v.7.13.6) [43]. We coded five (types of) events (see table 3 and figure 3): latency to leave the start box (for habituation trials 2 and 3, as well as the six test trials), persisting (test trials only), moment of detouring the barrier (test trials only), interacting with the food bowl (for habituation trials 2 and 3, as well as the six test trials) and leaving the species-specific ‘test box zone of interest’ (test trials only). All videos were coded by the first author. A second person blind to the hypotheses coded 10% of the videos per species. An average Cohen’s Kappa [44] was calculated for these videos to provide a measure of inter-rater, cross-species reliability. We had registered that, in the case no perfect inter-rater, cross-species agreement (0.81 ≤ Cohen’s Kappa ≤ 1) had been reached, discrepancies in inter-rater reliability would be investigated by calculating the average Cohen’s kappa [44] for each species, separately. By doing so, a species-specific or overall low Cohen’s Kappa would reveal whether the videos have to be recoded for one or all four species. However this was not needed, as the average cross-species Cohen’s kappa value indicated a strong level of inter-rater, crossspecies agreement (k = 0.927; [44]).

**Table 3.** The description of the behaviours that were coded in BORIS.

behaviour	description
leaving start box	- when the bird voluntarily leaves the start box: when both feet of the bird are visibly inside the test box, or (when the feet are not visible) when the front body half of the bird is inside the test box. - when the bird needs to be pushed: When the bird's entire body is inside the test box.^a^
persisting	at least the bird's whole head crosses the (fictitious) lines of the rectangular-shaped, species-specific ‘barrier zone of interest’.^b^
detour	at least the bird's whole head crosses the (fictitious) straight line from the barrier to the side of the test box (with a modifier whether they detour on the right or left side)
interaction with food bowl	bird touches the food or food bowl with its beak.
leaving the 'test box zone of interest'	at least the bird's whole head crosses the (fictitious) straight line at ≈ 2/3 of the test box length.^b^

^a^ When a bird needed to be gently pushed, it was most likely that the individual was lying down in the startbox. As a result, a gentle push put the bird forward and resulted in the bird ending up in a standing position in the test box. As a result, we coded whether these individuals had left the start box when the bird’s entire body (vs. front half body) was inside the test box.

^b^ The fictitious lines that marked a zone of interest were defined by two wooden sticks attached to each side of the test box.

To compare detour performance between species, we extracted our two response variables from the behavioural events coded in BORIS. First, the latency to detour (in seconds) was determined as the time between leaving the start box and the moment the individuals detoured the barrier. A maximum trial duration of 135 seconds for detour latency was assigned to the 20 trials (1.40% of the dataset) in which birds did not detour but entered the species-specific ‘barrier zone of interest’. Second, the time spent persisting (in seconds) was calculated as the cumulative time that the individual spent in the species-specific ‘barrier zone of interest’ (size = Barrier L × 25% of the Barrier-Entry Distance; L × W, see table 2 for the species-specific dimensions). A minimum trial duration of 0 seconds for persisting was assigned to the 483 trials (33.82% of the dataset) in which birds detoured without entering the species-specific ‘barrier zone of interest’ first. Third, a ‘multi-baseline’ measure of an individual’s general motivational state (in seconds) was calculated, by *averaging* the time between leaving the start box and touching the food (bowl) placed in front of the opaque barrier on habituation trials 2 and 3. Note that if a bird was unsuccessful on trial 2, a non-averaged ‘multi-baseline’ score was calculated based on habituation trial 3 only.

### 6.2. Data exclusion criteria

Individuals that failed to visit the food bowl at the third habituation day were excluded from subsequent test trials (pre-test criterion). This exclusion criterion guaranteed a similar within- and between-species proficiency with the basic task demands (e.g. the perceptual, motoric and motivational requirements to retrieve a food reward; for a similar pre-test exclusion criterion see [45]).

Birds that did not detour around the barrier nor entered the species-specific ‘barrier zone of interest’ in a test trial were excluded from subsequent test trials (and data of that individual was excluded from all statistical analyses). This mid-test exclusion criterion 1 was applied for two reasons. First, birds that did not obtain a measure for one of the two dependent variables within 2 minutes were likely to be unmotivated or be in distress. Furthermore, observations from similar RI test paradigms in our laboratory demonstrate that such individuals are unlikely to eat at all with a prolonged test time or on subsequent test trials (within the same day).^10^ In addition, removing birds from subsequent trials (rather than assigning a maximum trial limit for both dependent variables) reduced the risk of data skewing.

Individuals that left the species-specific ‘test box zone of interest’ (size = 2 times the Barrier-Entry Distance, see table 2 for the species-specific dimensions) without touching the food (bowl) were also excluded from further testing and all analyses. This mid-test exclusion criterion 2 ensured that we avoided confusing general exploration behaviour (without initial interest in the food) with successful detour performance (which assumes interest in the food). Thus, by excluding birds with differential trial experiences (due to for example demotivation, distress, distraction or exploration; for a similar mid-test exclusion criterion see [15]), we aimed to ensure that each barrier orientation is standardized within and between species. We registered that we would test all individuals of each species in a single ‘season’, as we incubated per season 20% more eggs than the number of individuals required for the testing; we expected that this surplus would allow us to replace all excluded individuals. For an overview of the birds excluded per criterion, see table 4. However, due to the fearful and stress responses of gulls during testing (we come back to this in the general discussion), the exclusion rate was higher than expected so we had to include a second breeding season.

**Table 4.** Number (and %) of birds excluded per criterion.

species	white leghorn chicken	Japanese quail	herring gull	fife fancy canary	total
initial sample	90	85	147	170	492
excluded for:					
pre-test criterion	10 (11%)	2 (2%)	35 (24%)	1 (1%)	48 (10%)
mid-test criterion 1	2 (2%)	4 (5%)	39 (26%)	14 (8%)	59 (12%)
mid-test criterion 2	7 (8%)	21 (25%)	0 (0%)	31 (18%)	59 (12%)
technical issues/sick birds	2 (2%)	0 (0%)	0 (0%)	9 (5%)	11 (2%)
remaining sample	69 (77%)	58 (68%)	73 (50%)	115 (68%)	315 (64%)

All raised birds were subjected to habituation and (part of) testing. As can be seen, the total number of birds tested was higher thanregistered for all species (apart from the quails). This was due to the fact that these individuals were reused for other studies, with differentsample size requirements. Reusing individuals in other behavioural studies is possible when they share similar prior experiences [11] and facilitates future analyses, such as exploring correlations between different tasks and making comparisonsacross studies.The first 60 individuals (58 for quails) that did not fail any exclusion criteria were selected for this study, ensuring a balanceddesign and minimizing group variation.

### 6.3. Statistical analysis

Statistical analyses were performed using R. v. 4.2.2 [46]. Models were fitted by means of the *lme4* package [47] and parameter estimation and p-values for the generated models were provided by means of the *carData* [48] and *car* [49] packages, which are suited for both linear mixed models (LMM) with temporal correlation structures and generalized linear mixed models (GLMM). For the LMM, we used partial eta-squared ($\eta_{p}^{2}$) as effect sizes for the relevant statistical models (linear mixed model) and they were calculated by means of the *effectsize* [50] package.

### 6.4. Registered model

We registered that we would perform a (G)LMM with Type III sum of squares on the latency to detour and the cumulative time spent in the species-specific ‘barrier zone of interest’ (persisting). These registered models (model specification 1) included the between-species factor: *Species* (i.e. chickens, quails, gulls and canaries) and both within-species factors: *Barrier* (i.e. VB and HB) and *Trial* (i.e. 1−3), and their interactions. In addition, they included two extra explanatory variables: a ‘*multi-baseline’* measure of an individual’s general motivational state (and its interaction with *Species*, as we mean-centred this ‘*multi-baseline’* measure within *Species*, see [51] for an example of within-group centring); and *Barrier Order* (with two levels: the individual received the HB barrier on the first test day 1 and the VB barrier on the second test day; or vice versa), as species might demonstrate superior performance with the last encountered barrier, irrespective of its type and ecological validity. Bird identity and enclosure (social group) were included as a random intercept in the models, with bird identity nested in enclosures. In addition, we included by-individual (nested in enclosures) random slopes that varied for the levels of *Species* (corresponding with species-specific intercepts). The registered model is presented in model specification 1.


(6.1) $$\begin{array}{rl} \text{Log(Outcome, s)}\;∼\; & \text{Species}\times (\text{Barrier}\times \text{Trial}+\text{Baseline})\\ & +\text{BarrierOrder}+(\text{Species}|\text{Id:Enclosure}) \end{array}$$

We registered that we would generate plots by means of the package *performance* [52] to inspect for violations of the model assumptions: (i) heteroscedasticity (plotting the square root of the residuals (y-axis) and fitted values (x-axis)), (ii) non-normality of residuals (plotting the sample quantiles (y-axis) on the standard normal distribution quantiles) and (iii) outliers (plotting standard residuals (y-axis) and leverage). Additionally, the multicollinearity between fixed main factors (via the variance inflation factor, VIF) and the autocorrelation between residuals (via a Durbin-Watson-Test) would be calculated via functions provided by the *performance* [52] package. Potential violations of model assumptions would be addressed by transforming the (in)dependent variables (i.e. via log-transformation) or by changing the error distribution (family) or the link function of the model (switching a default LMM that will be fitted to a GLMM). Fixed main effects with a VIF of >5 were planned to be removed and logical outliers (i.e. recording/entry errors) would be inspected and corrected (if possible). In the case that the outlier could not be corrected, all data of that individual was planned to be excluded from all statistical analyses.

### 6.5. Applied model

Following the registered inspections and analyses, changes were made to the registered models to address model complexity, violations of certain assumptions, and issues with model convergence as these problems would undermine the validity of the original model’s outcomes and lead to misleading or unreliable results. The statistical inferences supporting these changes are provided in the electronic supplementary materials [35].

For detour latency, the registered model (model specification 1) was simplified by removing the random slope for Species in order to reduce the model’s complexity. This decision was based on the presence of perfect or near-perfect correlations among random effects, indicating redundancy and boundary singularity (see electronic supplementary material, table S1, [35]). In order to address violations of the model assumptions (i.e. heteroscedasticity and non-normality of residuals), the dependent variable was log-transformed (see electronic supplementary material, figure S1, [35]). To address autocorrelation in the residuals,^11^ the model was further extended with a temporal correlation structure using the *nlme* package [53]. This temporal correlation structure accounts for the correlation in residuals from repeated measurements across Time (i.e. 1−6 trials; for each bird, nested within enclosures). Specifically, each bird participated in two sessions, with one session per barrier type and three trials per session, resulting in six interdependent trials. The autocorrelation parameter (ϕ), estimated by the model at lag 1, was 0.319. Explicitly modelling this autocorrelation properly accounts for the residuals’ temporal dependencies (see electronic supplementary material, figure S2, [35]), leading to an improved model fit (AIC = 4063.716 with the correlation structure vs. AIC 4275.634 without) and more accurate parameter estimates (see electronic supplementary material, table S12, [35]). Adding this correlation structure was required as adjusting the error distribution (e.g. gamma or inverse Gaussian) did not resolve the autocorrelation issue, as the models with adjusted error distributions encountered convergence problems. VIF scores were all below 5, and no logical outliers were detected, so we did not have to remove any outliers. The applied model for detour latency is presented in model specification 2; for an overview of the evolution of the model structure, see electronic supplementary materials [35].


(6.2) $$\begin{array}{rl} \text{Log(Detour Latency, s)}\;∼\; & \text{Species}\times (\text{Barrier}\times \text{Trial}+\text{Baseline})+\text{BarrierOrder}+(1|\text{Id:Enclosure})\\ & +\text{corAR1}(\text{Time}|\text{Id:Enclosure}) \end{array}$$

For persisting, the registered model (model specification 1) was simplified by removing the random slope for Species, for the same reasons as in the statistical model for detour latency (see electronic supplementary material, table S2, [35]). The simplified model demonstrated violations of model assumptions (i.e. heteroscedasticity and non-normality of residuals; see electronic supplementary material, figure S3, [35]), which could not be addressed by log-transforming the dependent variable due to the presence of zeros in the data. To meet model assumptions, various models with different error distributions were explored, including Poisson, negative binomial (ZI) and zero-inflated negative binomial (ZINB), with the selection guided by the data characteristics. The dependent variable, persisting, was therefore also converted to integer counts by scaling the original data to frames (30 frames per second), which was necessary to meet the model’s requirements while preserving the precision of short latencies that would otherwise be rounded to zero. The selected models are designed to account for overdispersion and excess zeros, reducing the need for explicit tests of heteroscedasticity and non-normality of residuals. However, additional diagnostics were conducted using the *DHARMa* package [54], assessing: (i) residual uniformity (Kolmogorov–Smirnov test), (ii) over/under-dispersion, (iii) outliers, (iv) zero-inflation, and (v) autocorrelation (via residual plots ^11^). Ultimately, the ZINB model, implemented via the *glmmTMB* package,^12^ satisfied the final model assumptions (see electronic supplementary material, figure S4, [35]). The ZINB model included a negative binomial component to capture variability in persisting and a zero-inflated part to account for the excess of zeros in persisting. The best-fitting zero-inflation component was determined using AIC comparisons across models with different combinations of main and interaction effects in the zero-inflated model’s formula, which resulted in the inclusion of *Barrier*, *Baseline*, *Species*, *Trial* and the interaction between *Species* and *Trial* as zero-inflated effects. VIF scores were all below 5, and no logical outliers were detected. The applied model for persisting is presented in model specification 3, see supplementary materials [35].


(6.3) $$\begin{array}{rl} \text{NB2(Persisting,frames)}\; & ∼\;\text{Species}\times (\text{Barrier}\times \text{Trial}+\text{Baseline})+\text{BarrierOrder}+(1|\text{Id:Enclosure})+\\ & \text{zi}∼\text{Barrier}+\text{Baseline}+\text{Species}+\text{Trial}+\text{Species:Trial} \end{array}$$

We had preregistered that, in the case of (i) significant *Barrier* × *Species* interaction effect(s) (*Prediction 1*), (ii) a main effect of *Trial* (*Prediction 2*), or (iii) a significant three-way *Species* × *Barrier* × *Trial* interaction effect(s) (*Explorative Prediction 3*), follow-up tests would be performed. We only found a significant effect of *Trial* (*Prediction 2*). In line with the preregistration, we performed *post hoc* Bonferroni–Holm corrected [58] linear contrasts upon the model to compare performance over trials (within one session). Follow-up linear contrasts along with the corresponding effect sizes (observed Cohen’s *d* for LMMs, Incidence Rate Ratio for the negative binomial part of the ZINB model and Odds Ratios for the zero-inflated part of the ZINB model) were calculated by means of the *emmeans* [59] and *lsr* (Cohen’s *d* [60]) packages.

## 7. Results

### 7.1. Detour latency

#### 7.1.1. Registered comparisons with the applied model

**Figure 4.**
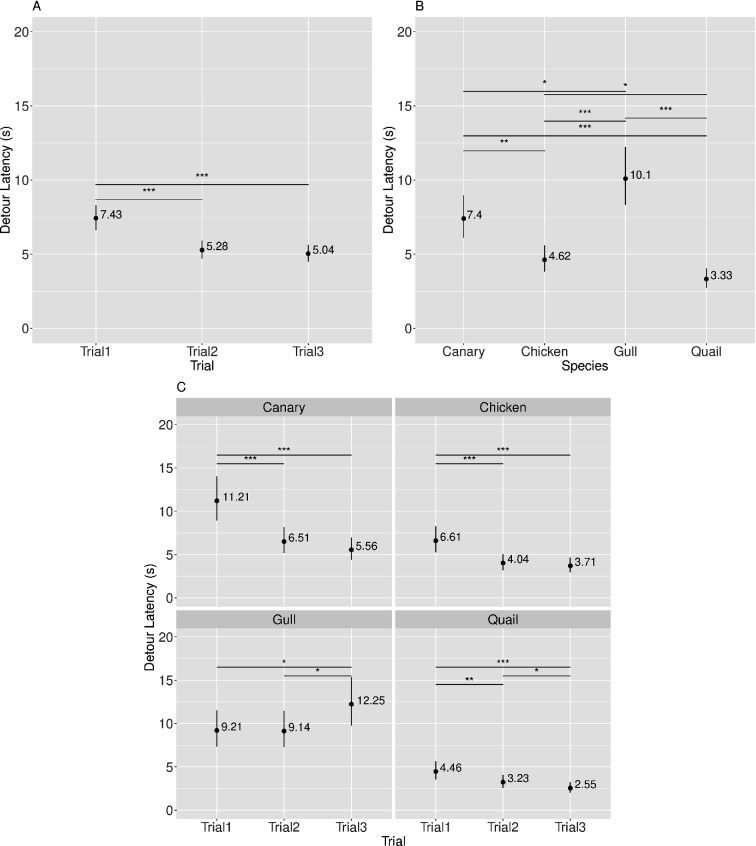
Visualization of model-predicted means (back-transformed to detour latency in seconds) along with their 95% CI across different Trial levels (A), Species (B) and Species by Trial interactions (C). Significant effects are indicated as follows: *p < 0.05, **p < 0.01, ***p < 0.001.

Descriptive statistics appear in figure 4 and tables 5–7; inferential statistics appear in table 8 and electronic supplementary material, tables S3–S5, [35]. The Species × Barrier (*Prediction 1*) and the Species × Barrier × Trial (*Prediction 3*) interaction effects were not significant. However, there was a significant main effect of Trial (*Prediction 2*), as shown in table 8.

**Table 5.** The model predicted means (on the log scale), the back-transformed model-predicted means (on the original scale, in seconds) and the observed means (also on the original scale, in seconds) for detour latency across different Trial levels.

	model		exp (model)		observed
**trial**	**mean (SE**)	**CI**	**mean**	**CI**	**mean (SD**)
trial1	2.005 (0.057)	1.892–2.118	7.428	6.635–8.316	15.276 (22.114)
trial2	1.664 (0.058)	1.550–1.777	5.279	4.712–5.914	11.636 (21.879)
trial3	1.617 (0.057)	1.504–1.730	5.038	4.500–5.640	10.418 (18.523)

**Table 6.** The model predicted means (on the log scale), the back-transformed model-predicted means (on the original scale, in seconds) and the observed means (also on the original scale, in seconds) for detour latency across different Species levels.

	model		exp (model)		observed
species	mean (SE)	CI	mean	CI	mean (SD)
canary	2.002 (0.097)	1.810–2.194	7.404	6.110–8.972	14.591 (22.203)
chicken	1.531 (0.097)	1.339–1.724	4.625	3.817–5.604	8.245 (13.867)
gull	2.313 (0.097)	2.121–2.505	10.100	8.335–12.239	18.517 (27.911)
quail	1.202 (0.099)	1.007–1.397	3.327	2.736–4.044	8.280 (14.771)

**Table 7.** The model predicted means (on the log scale), the back-transformed model-predicted means (on the original scale, in seconds) and the observed means (also on the original scale, in seconds) for detour latency across different Trial nested within Species levels.

	model		exp (model)		observed
condition	mean (SE)	CI	mean	CI	mean (SD)
canary					
trial1	2.417 (0.114)	2.192–2.642	11.209	8.952–14.035	20.890 (26.208)
trial2	1.874 (0.115)	1.647–2.100	6.511	5.193–8.165	13.992 (24.149)
trial3	1.716 (0.114)	1.491–1.941	5.561	4.441–6.963	8.892 (12.004)
chicken					
trial1	1.888 (0.114)	1.664–2.113	6.608	5.278–8.274	11.423 (17.141)
trial2	1.395 (0.115)	1.169–1.621	4.035	3.218–5.060	6.510 (9.563)
trial3	1.311 (0.114)	1.086–1.536	3.709	2.963–4.644	6.801 (13.399)
gull					
trial1	2.220 (0.114)	1.995–2.445	9.206	7.352–11.527	16.243 (22.341)
trial2	2.213 (0.115)	1.986–2.439	9.140	7.289–11.461	18.424 (31.530)
trial3	2.505 (0.114)	2.280–2.730	12.247	9.781–15.335	20.885 (29.086)
quail					
trial1	1.496 (0.116)	1.268–1.725	4.465	3.552–5.612	12.452 (20.766)
trial2	1.174 (0.117)	0.944–1.404	3.234	2.569–4.071	7.479 (12.313)
trial3	0.936 (0.116)	0.707–1.164	2.549	2.028–3.204	4.909 (6.770)

**Table 8.** Output: LMM with temporal correlation structure on detour latency (s).

parameter	${\tilde{X}}^{2}$	Df	P	*np* ^2^
(intercept)	1322.498	1	**<0.001**	
species	77.015	3	**<0.001**	0.249
barrier	1.343	1	0.246	0.001
trial	64.249	2	**<0.001**	0.051
baseline_centred	0.568	1	0.451	0.000
barrierorder	2.852	1	0.091	0.013
barrier:trial	0.151	2	0.927	0.000
species:barrier	0.147	3	0.986	0.000
species:trial	56.035	6	**<0.001**	0.045
species:baseline_centred	5.452	3	0.142	0.023
species:barrier:trial	8.228	6	0.222	0.007

Significant effects are indicated with bold *p-values.*

Follow-up contrasts upon the model for the main effect of Trial showed that performance improved over trials, with significantly slower detour latencies on Trial 1 compared to Trials 2 and 3. There was no significant difference in detour latencies on Trial 2 compared to Trial 3 (see table 5 and figure 4A). Further inferential statistics are provided in the electronic supplementary material, table S3.

#### 7.1.2. Additional exploratory analyses

Further examination of the model revealed an unexpected significant main effect of Species (see table 8). All pairwise comparisons were statistically significant (see table 6 and figure 4B). Further inferential statistics are provided in the electronic supplementary material, table S4.

There was also an unexpected interaction between Species × Trial (see table 8). *Post hoc* linear contrasts showed that performance improved for canaries, chickens and quails, but not for gulls. Specifically, Quails exhibited slower detour latencies on Trial 1 compared to Trials 2 and 3. Quails were also significantly faster on Trial 3 than Trial 2. Canaries and chickens exhibited slower detour latencies on Trial 1 compared to Trials 2 and 3. There was no significant effect between Trials 2 and 3 for either species. For gulls detour latencies were significantly faster on Trial 1 compared to Trial 3. Similarly, detour latencies on Trial 2 were significantly faster than on Trial 3. No significant effect was observed between Trials 1 and 2 (see table 7 and figure 4C). Further inferential statistics are provided in the electronic supplementary material, table S5.

### 7.2. Persisting

#### 7.2.1. Registered comparisons with the applied model

**Figure 5.**
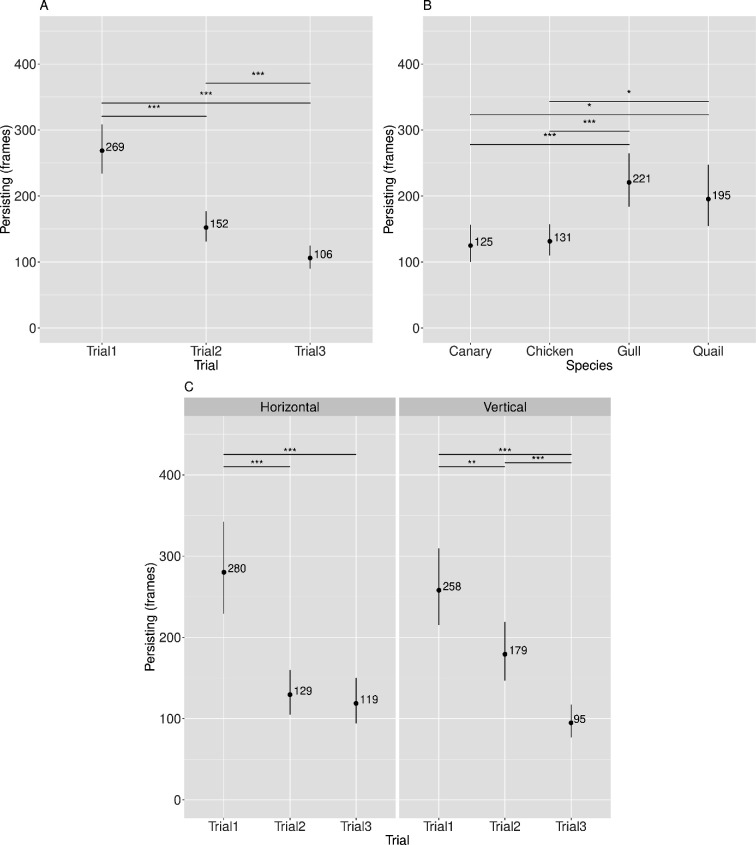
Visualization of the negative binomial model-predicted means (back-transformed log estimates to the original scale, representing, persisting in frames) along with their asymptotic CI across different Trial levels (A), Species (B) and Trial by Barrier interactions (C). Significant effects are indicated as follows: *p < 0.05, **p < 0.01, ***p < 0.001.

Descriptive statistics for the negative binomial part of the model appear in figure 5 and tables 9–11; inferential statistics appear in table 12 and electronic supplementary material, tables S6–S8 [35].

**Table 9.** The model predicted means (on the log scale), the back-transformed model-predicted means (on the original scale, in frames) and the observed means (also on the original scale, in frames) for persisting across different Trial levels.

	model		exp (model)		observed
trial	mean (SE)	CI	mean	CI	mean (SD)
trial1	5.594 (0.070)	5.456–5.732	268.769	234.144–308.514	295.861 (389.194)
trial2	5.025 (0.077)	4.875–5.176	152.233	130.941–176.987	204.441 (396.460)
trial3	4.664 (0.083)	4.501–4.827	106.037	90.095–124.800	148.066 (288.298)

**Table 10.** The model predicted means (on the log scale), the back-transformed model-predicted means (on the original scale, in frames) and the observed means (also on the original scale, in frames) for persisting across different Species levels.

	model		exp (model)		observed
species	mean (SE)	CI	mean	CI	mean (SD)
canary	4.827 (0.114)	4.604–5.051	124.886	99.898–156.123	190.718 (337.446)
chicken	4.878 (0.092)	4.698–5.059	131.418	109.747–157.368	152.094 (171.974)
gull	5.396 (0.093)	5.214–5.579	220.627	183.838–264.778	305.363 (505.505)
quail	5.275 (0.120)	5.039–5.511	195.420	154.331–247.448	238.181 (345.849)

**Table 11.** The model predicted means (on the log scale), the back-transformed model-predicted means (on the original scale, in frames) and the observed means (also on the original scale, in frames) for persisting across Trial nested in Barrier levels.

	model		exp (model)		observed
condition	mean (SE)	CI	mean	CI	mean (SD)
horizontal					
trial1	5.635 (0.102)	5.435–5.835	279.997	229.293–341.914	18.442 (23.536)
trial2	4.862 (0.107)	4.653–5.072	129.346	104.915–159.466	14.458 (24.518)
trial3	4.776 (0.119)	4.543–5.009	118.610	93.968–149.715	16.337 (26.444)
vertical					
trial1	5.553 (0.092)	5.372–5.734	257.991	215.250–309.218	19.378 (24.638)
trial2	5.188 (0.102)	4.989–5.388	179.170	146.774–218.717	16.757 (26.925)
trial3	4.552 (0.108)	4.340–4.763	94.797	76.744–117.097	12.214 (17.545)

**Table 12.** Output: GLMM on persisting (frames). Significant effects are indicated with bold p-values.

parameter	${\tilde{X}}^{2}$	Df	p
	negative binomial part		
(intercept)	9266.680	1	**<0.001**
species	24.031	3	**<0.001**
barrier	0.005	1	0.942
trial	97.222	2	**<0.001**
baseline_centred	1.506	1	0.220
barrierorder	2.302	1	0.129
barrier:trial	8.514	2	**0.014**
species:barrier	5.292	3	0.152
species:trial	3.949	6	0.684
species:baseline_centred	4.200	3	0.241
species:barrier:trial	4.150	6	0.656

	zero-inflated part		
(intercept)	153.731	1	**<0.001**
barrier	11.758	1	**<0.001**
baseline_centred	12.733	1	**<0.001**
species	174.552	3	**<0.001**
trial	35.177	2	**<0.001**
species:trial	12.573	6	0.050

**Figure 6.**
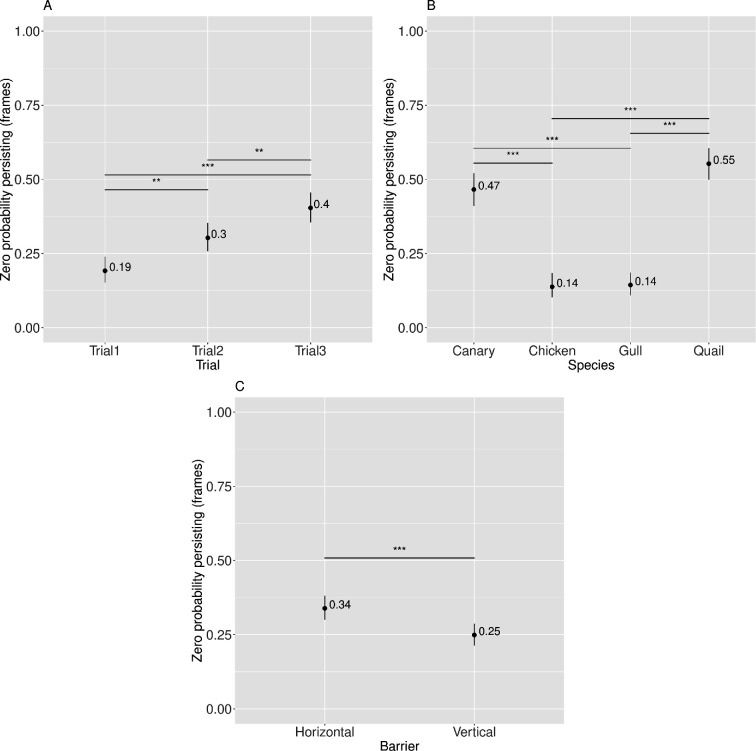
Visualization of the zero-inflated model-predicted means (back-transformed to the probability zeros for persisting in frames) along with their asymptotic CI across different Trial (A), Species (B) and Barrier levels (C). Significant effects are indicated as follows: *p < 0.05, **p < 0.01, ***p < 0.001.

**Table 13.** The model predicted means (on the log odds ratio scale), the back-transformed zero probabilities (on the original scale, in frames) and the observed zero probabilities (also on the original scale, in frames) for persisting across Trial levels.

	model		prob (model)		observed
trial	mean (SE)	CI	prob	CI	prob
trial1	−1.438 (0.143)	−1.718 to −1.158	0.192	0.153 to 0.239	0.244
trial2	−0.832 (0.116)	−1.058 to −0.605	0.303	0.258 to 0.353	0.342
trial3	−0.388 (0.107)	−0.597 to −0.180	0.404	0.355 to 0.455	0.429

**Table 14.** The model predicted means (on the log odds ratio scale), the back-transformed zero probabilities (on the original scale, in frames) and the observed zero probabilities (also on the original scale, in frames) for persisting across Species levels.

	model		prob (model)		observed
species	mean (SE)	CI	prob	CI	prob
canary	−0.139 (0.114)	−0.362 to 0.085	0.466	0.410 to 0.521	0.478
chicken	−1.831 (0.175)	−2.173 to −1.489	0.138	0.102 to 0.184	0.169
gull	−1.785 (0.157)	−2.093 to −1.477	0.144	0.110 to 0.186	0.158
quail	0.211 (0.111)	−0.006 to 0.428	0.553	0.499 to 0.605	0.555

**Table 15.** The model predicted means (on the log odds ratio scale), the back-transformed zero probabilities (on the original scale, in frames) and the observed zero probabilities (also on the original scale, in frames) for persisting across Barrier levels.

	model		prob (model)		observed
barrier	mean (SE)	CI	prob	CI	prob
horizontal	−0.665 (0.092)	−0.846 to −0.485	0.339	0.300 to 0.381	0.377
vertical	−1.106 (0.100)	−1.302 to −0.910	0.249	0.214 to 0.287	0.300

**Table 16.** The model predicted means (on the log odds ratio scale), the back-transformed zero probabilities (on the original scale, in frames) and the observed zero probabilities (also on the original scale, in frames) for the average value of Baseline.

	model		prob (model)		observed
average baseline value	mean (SE)	CI	prob	CI	prob
0	−0.886 (0.071)	−1.026 to −0.746	0.292	0.264 to 0.322	0

The Species × Barrier (*Prediction 1*) and the Species × Barrier × Trial (*Prediction 3*) interaction effects were not significant. However, there was a significant main effect of Trial (*Prediction 2*), as shown in table 12.

Follow-up contrasts upon the negative binomial part of the model to further investigate the main effect of Trial showed that performance improved over trials, with significantly more persisting on Trial 1 compared to Trials 2 and 3. There was also significantly more persisting on Trial 2 compared to Trial 3 (see table 9 and figure 5A). Further inferential statistics are provided in the electronic supplementary material, table S6.

#### 7.2.2. Additional exploratory analyses

Further examination of the negative binomial part of the model revealed an unexpected significant main effect of Species (see table 12). *Post hoc* linear contrasts showed that Canaries and Chickens persisted less compared to Gulls and Quails. All pairwise comparisons were statistically significant, except for the comparisons between Canaries and Chickens, and between Gulls and Quails (see table 10 and figure 5B). Further inferential statistics are provided in the electronic supplementary material, table S7. The main effect of Species will be addressed further in the discussion.

There was also an unexpected interaction between Barrier × Trial (see table 12). *Post hoc* linear contrasts showed that overall performance improved with both types of barriers. For the HB barrier, individuals persisted significantly more on Trial 1 compared to Trials 2 and 3. There was no significant difference between Trails 2 and 3. For the VB barrier, individuals persisted significantly more on Trial 1 compared to Trials 2 and 3. In addition, there was significantly more persisting on Trial 2 compared to Trial 3 (see table 11 and figure 5C). Further inferential statistics are provided in the electronic supplementary material, table S8.

In addition to the negative binomial component, the statistical model for persisting also included a zero-inflated component that accounted for excess zeros in persisting (i.e. capturing all birds that did not persist). Descriptive statistics for the zero-inflated part of the model appear in figure 6 and tables 13, 14, 15 and 16; inferential statistics appear in table 12 and electronic supplementary material, tables S9–S11, [35].

Examination of the zero-inflation part of the model revealed a significant main effect of Trial (see table 12). *Post hoc* linear contrasts showed that overall there was an increase in the probability of zeros for persisting in later trials, indicating improved accuracy. Specifically, Trial 3 showed a significant higher probability of zeros for persisting compared to Trials 2 and 1. Trial 2 also had a significantly higher probability of zeros for persisting compared to Trial 1 (see table 13 and figure 6A). Further inferential statistics are provided in the electronic supplementary material, table S9.

Again, we had not predicted a main effect of Species (see table 12). However, canaries and quails had a higher probability of zeros for persisting (indicating higher accuracy) compared to chickens and gulls. All pairwise comparisons were statistically significant, exception for the comparisons between canaries and quails, and between chickens and gulls (see table 14 and figure 6B). Further inferential statistics are provided in the electronic supplementary material, table S10. The main effect of Species will be addressed further in the discussion.

There was also an unexpected significant main effect of Barrier (see table 12). *Post hoc* linear contrasts showed a significantly higher probability of zeros for persisting (indicating higher accuracy) for for the HB than VB barrier (see table 15 and figure 6C). Further inferential statistics are provided in the electronic supplementary material, table S11.

An unexpected main effect of Baseline was also observed (see table 12). Follow-up analyses indicated that the probability of zeros persisting was estimated at 0.292, when birds had an average motivation score (Baseline at zero, due to within-species mean-centring). Descriptive statistics appear in table 16; inferential statistics appear in table 12.

### 7.3. Additional analysis: group size as a random effect

Due to post-hatch mortality (in canaries, gulls and quails), group size deviated slightly from the intended 10 individuals per group (table 17) as batch incubation limited the ability to replace these losses with age-matched individuals. Note that group sizes greater than 10 resulted from the merging of two high mortality enclosures where birds were of the same age. For the canaries, this was further complicated by the need to introduce ‘tutors’ for the juveniles (i.e. adult demonstrators to teach independent feeding), which meant that each group of juveniles (about 10) had at least one extra adult for a few days. To examine the possible impact of variation in group size on performance, additional analyses were conducted using ‘Group Size’ as a random variable. For both detour latency and persistence, the extended model did not outperform the above-mentioned reported models. As a result, these additional analyses are discussed in the electronic supplementary materials (tables S12 and S13).

**Table 17.** Visualization of the number of individuals that met our exclusion criteria, in relation to the enclosure group size and the species.

	group size								
species	#6	#8	#9	#10	#11	#12	#14	**mean**	range
canary	0	0	15	20	14	0	11	10.717	9–14
chicken	0	0	0	60	0	0	0	10	10–10
gull	0	22	3	35	0	0	0	9.217	8–10
quail	3	2	7	39	0	7	0	9.845	6–12

## 8. Discussion

We argue that stop-signal detection is a critical cognitive component of RI across species, including birds. This study explored this idea further by investigating whether RI is improved when the perceptual characteristics of the stop signal (i.e. barrier) in the detour barrier task correspond to the species’ ecological niche, as shown by Regolin *et al.* [9] and Zucca *et al.* [12]. However, we failed to replicate this earlier work, as RI was not significantly improved when the barrier type supposedly matched the ecological niche of the species (*Prediction 1*). On the other hand, we did find that performance generally improved over trials (*Prediction 2*) for both detour latency and persisting, but again, this did not interact with the species-specific ecological validity of the stop signal (*Prediction 3*).

Most importantly, we were unable to replicate the finding that barrier type had a species-specific influence on detour performance, even though our study has several methodological and conceptual strengths, including a well-powered design, standardized experimental procedures, controlled prior experience (through pre- and mid-test exclusion criteria), and baseline measures (to minimize confounding by non-cognitive, motivational traits). Thus, our findings do not support the ecological-niche hypothesis as proposed by Regolin *et al.* [9] and Zucca *et al.* [12], suggesting that the adaptation to a specific ecological niche cannot account for variation in stop-signal detection (at least, not in the detour task). This does not necessarily imply that stop-signal detection is not important at all for RI, but it does indicate that differences between the four bird species tested here are not caused by variation in how they perceive or interpret VB and HB barriers.

As trials progressed, most individuals became faster in detouring (except for gulls) and made fewer attempts to persist in interacting with the barrier, regardless of the barrier type (confirming Prediction 2 but disconfirming Prediction 3). In the habituation set-up (or training phase) of our study, the food bowl was placed *in front* of the opaque barrier; this ensured that birds had no prior experience of retrieving food from behind a barrier (which standardized baseline performance). But without this experience, in the test phase, individuals had to learn both to inhibit their prepotent response to go directly for the reward (as the direct path is blocked) and to navigate around the barrier [10], explaining the observed improvements over trials.

The learning pattern observed for the gulls was unexpected, as it appears that gulls learned to inhibit interacting with the barrier itself but without an overall improvement in detour latency, whereas the other species became faster at detouring and interacted less over time with the barrier. At present, we have no explanation as to why, for gulls, learning was only observed for the persistence measure and not detour latency as was seen in the other three species. However, this pattern demonstrates the value of looking at detour latency and time spent interacting with the barrier. One might assume that lower persistence scores should automatically result in shorter detour latencies but for gulls, this was not the case. This indicates that overall task performance (i.e. detour latency) captures additional behaviours, potentially unrelated to RI (e.g. the time taken to approach the barrier, time spent not interacting, time needed to navigate the barrier, etc.). The observed differences in learning also highlight two further issues. First, the fact that gulls showed evidence of learning in measures of persistence but not in the measure of detour latency suggests that, at least for some species, tasks include several subcomponents and that some of these are not equally influenced by learning across species; certain task components are more influenced by learning (inhibiting an unrewarded repetitive response) than others (inhibiting the response to go straight for the food or navigating around a barrier, which are both captured by detour latency). Speculatively, this may relate to adaptations to the species’ ecological niches. Inhibition of unrewarded responses is likely to be a critical component of adaptive behaviour across the different ecological niches experienced by the species tested here, and therefore more easily learned by all species. In contrast, navigating obstacles may depend more on context-specific factors, such as available navigational cues and spatial scale. This may make learning more challenging for some species, especially if the test environment does not match their ecological niche. For example, while gulls may excel at using large-scale spatial cues in open spaces, they may struggle with small-scale obstacles in confined environments such as a test box. However, more research is needed to explore this idea. Second, the learning differences stress the need to take the role of learning in RI (and cognition in general) into account when aiming to interpret the variation in RI between species. For example, while canaries and gulls were initially slow at detouring (compared to chickens and quails; figure 4), detour latencies of canaries gradually decreased, while those of gulls did not. This suggests that the differences between these two species in a putative test of RI could at least partly reflect variations in learning rather than inhibition, with interaction effects between species and trial potentially explaining these findings [61].

Alongside the effects of trial, we also identified general latency differences between species. Even though such differences are hard to interpret, one notable finding stands out, namely that gulls appeared to ‘underperform’ compared to the other species, as they were generally slower (compared to the three other species; figure 4), more likely to peck (compared to canaries and quails; figure 6), and when they did peck, they pecked for longer (compared to canaries and chickens; figure 5). As noted above, the gulls’ detour latencies also did not decrease over trials. We consider two (not mutually exclusive) hypotheses. First, the gulls are a wild species, whereas the three other species are domesticated. Domesticated species are generally less fearful and stressed than wild species [62]. For example, Gjøen *et al.* [63] compared the behavioural responses of white leghorn chickens with their wild counterparts, red junglefowls (*Gallus gallus*), in risk-taking situations, such as the encounter of a novel object during food retrieval. They found that red junglefowls were more stressed and fearful of the object and reached the food later than white leghorns. If gulls were indeed more fearful and stressed than the other species, this could have influenced their detour performance. Consistent with this idea, there was a high number of drop-outs among gulls (compared to the three other species; table 4) due to the pre-test (i.e. a failure to interact with the food bowl in the presence of a novel barrier in a new test environment) or mid-test 1 (i.e. a failure to obtain a measure for one of the two dependent variables during a test trial, indicating little interaction with the experimental task) exclusion criteria. Second, even though we standardized the testing age in terms of number of days, the developmental trajectories of RI (and cognition in general) may have differed between species. Gulls have a much longer maximum lifespan (49 years) compared to canaries (24 years), chickens (15–20 years) and quails (6 years [64]); based on life-history theories, one could speculate that neuro-cognitive development would be protracted in the semi-precocial and long-living gulls compared with for example the precocial and shorter-living quails and chickens [65]. However, this idea should be further tested.

Finally, it is noteworthy that canaries successfully solved (and learned) the detour problem (irrespective of barrier type). In contrast, in the study of Zucca *et al.* [12], canaries were unable to solve the detour problem and repeatedly attempted to fly over the barrier (again, irrespective of barrier type). The authors attributed this inability to the canaries’ adaptation to an aerial environment, which enables them to navigate obstacles by simply flying over them in natural environments. However, several other studies have already shown that species, adapted to an aerial lifestyle, such as ravens [6], ring doves (*Streptopelia risoria* [7,66]) and pigeons (*Columbia livia*, [7,66]), are capable of solving the detour barrier task as well. We speculate that canaries were able to solve the detour problem in our study, but not in the original work, due to the exclusion criteria we implemented, which ensured proficiency with the basic task demands (e.g. the perceptual, motoric and motivational requirements for retrieving a food reward [45]). Specifically, our pre-test exclusion criterion ensured that all included birds visited and ate from a food bowl placed in front of a barrier (novel object) in the habituation phase before access to the food bowl was restricted by moving the barrier in front of it in the test phase. We believe that experience with retrieving the reward may be critical for measuring detour performance, potentially more so in aerially adapted birds. After all, Zucca *et al*. [12] found that, even after prolonged exposure to the test situation, a large proportion of canaries were unable to solve the detour problem. This suggests that the problem was not a lack of familiarity with the test itself, but rather a lack of experience with retrieving the reward. However, this explanation is speculative and requires further investigation.

In summary, we failed to provide support for the ‘ecological niche hypothesis’, as proposed by Regolin *et al*. [9] and Zucca *et al*. [12]. Our study adds to the growing body of evidence for the critical need for replication studies [33] and highlights the need to consider methodological and conceptual design factors, as these can significantly impact results. Although our study did not provide strong evidence for the idea that interspecies differences in the perception of barrier types influence detour performance (and cause species differences), this does not negate the need for further research into the influence of the characteristics of the stop signal or other underlying mechanisms of RI. More generally, future research should focus on the cognitive mechanisms underlying RI. Understanding these mechanisms will help explain inter-individual variation such as in decision-making in dynamic environments [4], predator avoidance and foraging optimization [5], as well as responses to broader ecological pressures [3].

## Data Availability

Data, script, codes and supplementary information are available at the OSF repository: [35]. Supplementary material is available online [67].

## Footnotes

^1^
Zucca et al. (2005) [12] mention in their paper that they tested herring gulls, but used the scientific name of the Caspian gull *Larus cachinnans*. The taxonomy of the genus Larus has been updated over the years. Given that the authors mentioned that the gulls were from a breeding colony in Trieste (Italy), we assume they tested yellow-legged gulls, which were formerly treated as a subspecies of the herring gull (as was the Caspian gull).

^2^
Zucca et al. [12] mention that they used a 23 × 26 cm barrier, barrier lines/rods with a width of 0.2 cm, and in-between line/rod gaps of 1.25 cm. Given these measurements, only a combination of 18 rods/lines and 18 in-between gaps is possible, which is the equivalent of a VB or HB barrier with a 14% reward occlusion.

^3^
Given the high availability of the herring gull at the North Sea coast (Belgium), herring gulls were selected to represent the gull species in this comparative study. But see also footnote 1.

^4^
The original studies administered a variety of barrier types, including a transparent barrier, which was then also used to interpret performance with the other barrier types. Yet, we opted to exclude transparent barriers from our design for three reasons. First, the comparison of transparent and barred barriers tests another hypothesis, namely the role of reward occlusion, rather than the ecological validity of the barrier type (which can only be tested by comparing VB and HB barriers). Second, performance in the detour task can be influenced by differential individual experiences with transparent obstacles [11,31]. Last, omitting the transparent barrier reduces the contact of wild gulls to humans, which is important as gulls are wild animals and will be raised as such, prior to their post-test release back to nature.

^5^
Farrar et al. [33] mention in their paper on replications in comparative cognition that power analyses are not the gold standard in this research domain, and ’in many cases comparative cognition researchers could be better off performing design or sensitivity analyses based on their resource constraints’.

^6^
This adjustment was made after data collection but prior to any data analysis. These changes are still considered part of Stage 1 IPA and were pre-approved.

^7^
This adjustment was made after data collection but prior to data analysis. These changes are still considered part of Stage 1 IPA and were pre-approved. Additionally, an analysis incorporating group size as an extra random variable in the statistical models is provided in the supplementary materials [35].

^8^
During the nestling period, birds were kept in cages with vertical wires (the default policy at the University of Antwerp). However, we believe that this does not affect the interpretation of our results. First the birds did not interact with the cage during the nestling period, so they could not learn whether the bars or strips were impenetrable or not. Second, Zucca *et al*. [12] argued that detour performance is improved when the perceptual characteristics of the stop signal match the ecological niche (rather than the prior experience) of the species. Notably, although the canaries in Zucca *et al*. [12] were reared in cages with vertical bars from two months until tested at four to six months, performance did not differ between barrier types.

^9^
Potential variations in colour perceptions and sensitivities across species are mitigated by the developed preference for their respective food bowls prior to the start of the experiment through repeated exposure (i.e. 10-day habituation to the food bowl in the enclosure) and learning (pairing of the coloured bowl with food during these 10 days).

^10^
In a continuous RI task with a sample size of 80 herring gulls, birds that failed on the first trial were likely to fail again on the second trial of the same test day (Dewulf, Knoch, *et al.,* 2025, unpublished data).

^11^
The Stage 1 registered Durbin-Watson statistic (using performance [52] package) and the ACF plot (using the base R package) are not suitable for (G)LMM (due to the random effect structure) an issue identified during the data analysis. Consequently, we opted for alternative methods to assess autocorrelation in the residuals. Specifically, we employed ACF plots (for LMM; using the package nlme [53]) or simulation-based residual plots (GLMM; using the package DHARMa, [54]) for inspecting autocorrelation between residuals.

^12^
The glmmTMB [55] package depends on both the Matrix [56] and TMB [57] packages. To fit a (ZI)NB model with random effects, it is essential to reinstall the Matrix [56] package from source (version 1.6.2) followed by reinstalling TMB [57] and GLMM TMB [55] from source.
